# Safety and efficacy of encorafenib–cetuximab combination in *BRAF*^*V600E*^-mutated metastatic colorectal cancer: real-world evidence from the CONFIDENCE Spanish multicenter study

**DOI:** 10.1016/j.esmorw.2024.100055

**Published:** 2024-07-16

**Authors:** A. Fernández-Montes, J. Ros, P. García-Alfonso, B. Graña, E. Brozos-Vázquez, M. Melián, A.Mª Jiménez-Gordo, E. Martínez de Castro, I. Busquier, A. Ferrández Arias, C. Grávalos, M. Covela, A. Ruíz-Casado, E. González-Flores, Mª J. Safont, J.Mª Cano, C. Alonso-López, Mª J. Gómez-Reina, O. Donnay, J.L. Manzano-Mozo, P. Sampedro Domarco, E. Falcó, N. Rodríguez, C. García-Benito, E. Aranda, Spanish Cooperative Group for the Treatment of Digestive Tumors (TTD)

**Affiliations:** 1 Department of Medical Oncology, Complejo Hospitalario Universitario de Ourense, Orense; 2 Department of Medical Oncology, Vall d’Hebron University Hospital and Vall d’Hebron Institute of Oncology (VHIO), Barcelona; 3 Department of Medical Oncology, Hospital Universitario Gregorio Marañón, Madrid; 4 Department of Medical Oncology, Complejo Hospitalario Universitario A Coruña, Instituto Investigación Biomédica INIBIC, La Coruña; 5 Department of Medical Oncology, Hospital Clínico Universitario de Santiago, Santiago de Compostela; 6 Department of Medical Oncology, Instituto Valenciano de Oncología, Valencia; 7 Department of Medical Oncology, Hospital Universitario Infanta Sofía, San Sebastián de los Reyes; 8 Department of Medical Oncology, Hospital Universitario Marqués de Valdecilla, Instituto de Investigación IDIVAL, Santander; 9 Department of Medical Oncology, Hospital Provincial de Castellón, Castellón; 10 Department of Medical Oncology, Hospital General Universitario de Elche, Elche; 11 Department of Medical Oncology, Hospital 12 de Octubre, Madrid; 12 Department of Medical Oncology, Hospital Lucus Augusti, Lugo; 13 Department of Medical Oncology, Hospital Universitario Puerta de Hierro, IDIPHISA, Majadahonda; 14 Department of Medical Oncology, Hospital Virgen de las Nieves, Instituto de Investigación Biosanitaria Ibs., Granada; 15 Department of Medical Oncology, Hospital General Universitario, CIBERONC, Universidad de Valencia, Valencia; 16 Department of Medical Oncology, Hospital General de Ciudad Real, Ciudad Real; 17 Department of Medical Oncology, Hospital General de Albacete, Albacete; 18 Department of Medical Oncology, Hospital Nuestra Señora de Valme, Dos Hermanas; 19 Department of Medical Oncology, Hospital Universitario de La Princesa, Madrid; 20 Department of Medical Oncology, ICO, Hospital Germans Trias i Pujol, Badalona; 21 Department of Medical Oncology, Hospital Son Llatzer, Palma de Mallorca; 22 Department of Medical Oncology, Hospital Universitario La Paz, Madrid; 23 Department of Medical Oncology, Hospital Meixoeiro-Hospital Álvaro Cunqueiro, Vigo; 24 Department of Medical Oncology, Hospital Universitario Reina Sofía, IMIBIC, Universidad de Córdoba, CIBERONC, Instituto de Salud Carlos III, Córdoba, Spain

**Keywords:** *BRAF*^*V600E*^-mutated metastatic colorectal cancer, cetuximab, encorafenib, real-world data, survival, safety

## Abstract

**Background:**

Dual blockade therapy encorafenib–cetuximab (EC) was recently established as the standard of care for second- or third-line treatment for *BRAF*^*V600E*^*-*mutated metastatic colorectal cancer (mCRC) patients based on BEACON phase III study results. The CONFIDENCE study aims to provide insight about the real-world (RW) safety and effectiveness of EC in a Spanish cohort.

**Materials and methods:**

This retrospective study included *BRAF*^*V600E*^-mutated mCRC patients treated in second line with EC in the RW setting. The primary endpoint (EC effectiveness) was measured by progression-free survival (PFS) and overall survival (OS). Key secondary endpoints were overall response rate (ORR), disease control rate (DCR), duration of response (DoR), potential factors affecting PFS and OS and safety.

**Results:**

Eighty-one patients were included after at least 5 months of follow-up before study onset, 50.6% female with a median age of 66.1 years. Overall, 65% of patients debuted with synchronous metastatic disease. Patients received a median of six EC cycles. The median OS and PFS after 9.7 months of follow-up were 12.6 and 5.0 months, respectively. The median DoR was 5.8 months. ORR was 33.8% and DCR was 68.8%. Alkaline phosphatase, neutrophil/lymphocyte ratio and three or more metastatic lesions were accurate prognostic factors for OS. Additionally, the presence of liver metastases has prognostic value for PFS. The most reported adverse events (AEs) were skin-related toxicities (43.2%). Grade ≥3 AEs occurred in 13.5% of patients.

**Conclusions:**

Our results align with the BEACON trial results, confirming the safety and efficacy of EC in the RW setting and additionally provide insight about survival prognostic factors.

## Highlights

• RW effectiveness and safety of EC align with data from clinical trials.
• Better survival outcomes may be potentially derived from early EC administration.
• Factors of poor prognosis need to be further investigated to reach consensus.

## Introduction

Colorectal cancer (CRC) is the third most frequent cancer in Western Europe and responsible for 9.4% of all-cause deaths worldwide.^1^ About 20% of new cases debut with distant metastases, and ∼50% of CRC will develop metastatic disease.^2^ The standard of care (SoC) for advanced CRC is based on doublet or triplet combinations of irinotecan, oxaliplatin and fluoropyrimidines (5-fluorouracil or capecitabine), or chemotherapy alone, or associated with targeted agents such as anti-vascular endothelial growth factor or anti-epidermal growth factor receptor therapy for RAS wild-type (WT) tumors.^3^^,^^4^ Even with the addition of targeted therapies, the current median overall survival (OS) remains poor, ranging from 25 to 30 months.^5^^,^^6^ Currently, the 5-year survival rate for metastatic CRC (mCRC) is ∼14%.^7^

The *BRAF*^*V600E*^ mutation is highly involved in the pathogenesis of mCRC and accounts for 5%-21% of mCRC.^8^, ^9^, ^10^, ^11^, ^12^, ^13^, ^14^, ^15^, ^16^, ^17^ It is more prevalent in older females and associated with right-sided primary tumor location, distant lymph node metastases and peritoneal spread.^17^^,^^18^ The presence of *BRAF*^*V600E*^ mutation is associated, among others, to a microsatellite instability phenotype and a worse OS, and therefore to a poorer prognosis at metastatic stage.^4^

To date, the BEACON study is the largest trial evaluating the dual combination encorafenib–cetuximab (EC) in *BRAF*^*V600E*^-mutated mCRC patients who progressed to one or two therapy lines. It reported a median OS of 9.3 months, a progression-free survival (PFS) of 4.3 months and an overall response rate (ORR) for patients with only one prior line of 20.0%. The median duration of response (DoR) was 5.5 months.^19^ These results led to the approval of EC as the current SoC for refractory *BRAF*^*V600E*^-mutated mCRC patients who have progressed to one or two lines.

The real-world data (RWD) study CONFIDENCE aimed to complement the BEACON trial results, providing data from a real-world (RW) effectiveness and safety obtained from a cohort of patients treated in routine clinical practice conditions. With this work we intend to provide fundamental information for health care professionals and scientific community, which may improve the treatment of mCRC *BRAF*^*V600E*^-mutated patients.

## Materials and methods

### Study design and patients

The CONFIDENCE study is an observational, retrospective, multicenter study collecting data from a *BRAF*^*V600E*^-mutated mCRC cohort of Spanish patients treated in second line with EC in the RW setting. The CONFIDENCE cohort population was selected following the criteria used in the BEACON study.^19^ Enrolled patients had a minimum follow-up of 5 months after treatment initiation (if alive). At study visit, patients were included, and investigators retrospectively collected their data from electronic medical records after requesting their written informed consent. A waiver of consent was considered for deceased patients or loss of follow-up. The study was conducted according to the Declaration of Helsinki and Spanish local regulations, and it was approved by the Ethics Committee of Galicia (Santiago de Compostela, Spain; protocol code TTD-21-01).

The primary endpoint was the RW effectiveness in terms of PFS and OS. Secondary endpoints included patient characteristics and disease profile before starting EC treatment, ORR, disease control rate (DCR) according to RECIST version 1.1, DoR, time to progression (TTP), potential factors affecting PFS and OS, safety profile, treatment sequence before and after second-line EC and EC exposure and management.

This study followed the Guidance for Reporting Oncology real-World evidence (GROW).^20^

### Statistical analysis

Descriptive data (demographic and clinical characteristics of patients, and the therapeutic sequence after EC treatment) were descriptively analyzed by measures of central tendency and dispersion, and counts and percentages. Time-to-event variables [PFS, OS, TTP, DoR and duration of disease control (DDC)] were analyzed using the Kaplan–Meier method, and presented along with 95% confidence interval (CI). Median OS was calculated from first EC administration to death by any cause, and median PFS was calculated from first EC administration to progression or death by any cause. TTP was calculated from first EC administration to date of disease progression. ORR was calculated as the percentage of patients achieving complete response (CR) or partial response (PR) as the best response to EC. DCR was calculated as the percentage of patients who achieved stable disease (SD), PR and CR as the best response to EC. DDC was calculated among patients achieving a CR, PR or SD from first documentation of PR, CR or SD after treatment initiation until progression or death by any cause. Multivariate Cox regression analysis of potential factors independently associated to PFS and OS was conducted, including variables with statistical significance *P* < 0.2 in the univariate Cox regression in a multivariate model with stepwise selection method. Hazard ratio and the 95% CIs were calculated.

## Results

### Patient disposition and characteristics

From March to July 2022, 84 patients were consecutively identified and after excluding 3 patients due to non-fulfilment of eligibility criteria, a total of 81 patients were considered assessable. Baseline demographic and clinical characteristics are presented in Table 1.

**Table 1:** Baseline demographic and clinical characteristics and treatments before EC

Patient and disease characteristics	*N* = 81
Female sex, *n* (%)	41 (50.6)
Age, median (IQR), years	66.1 (58.9-71.2)
Tumor stage at diagnosis according to TNM staging system,^a^*n* (%)	
II	7 (8.8)
III	21 (26.3)
IV	52 (65.0)
Location of primary tumor, *n* (%)	
Left sided	31 (38.3)
Right sided	48 (59.3)
Both left sided and right sided	2 (2.4)
ECOG performance status at study entry,^b^*n* (%)	
0	28 (36.8)
1	42 (55.3)
2	6 (7.9)
Metastases’ locations at study entry, *n* (%)	
Liver	44 (54.3)
Lymph nodes	30 (37.0)
Peritoneum	29 (35.8)
Lung	22 (27.2)
≥3 metastases sites at study entry, *n* (%)	18 (22.2)
Time from metastatic diagnosis to EC onset, median (IQR), months	9.0 (6.1-16.0)
**Targetable molecular alterations,**^c^***n* (%)**	***N* = 81**
*KRAS* mutated	1 (1.4)
*NRAS* mutated	1 (1.4)
MSI-H, *n* (%)	8 (12.1)
*HER2* amplified, *n* (%)	
IHC positive	1 (14.3)
IHC negative	5 (71.4)
NGS negative	1 (14.3)
**CRC treatments before EC initiation**	
**Adjuvant/neoadjuvant (*n*)**	23
Chemotherapy-based fluoropyrimidine	5 (21.7)
Chemotherapy-based oxaliplatin	18 (78.3)
**First line (*n*)**	81
Chemotherapy based, *n* (%)	27 (33.3)
Chemotherapy-based fluoropyrimidine	4 (4.9)
Chemotherapy-based oxaliplatin	10 (12.3)
Chemotherapy-based irinotecan	10 (12.3)
Chemotherapy-based irinotecan and oxaliplatin	3 (3.7)
Chemotherapy based and antiangiogenic, *n* (%)	53 (65.4)
Bevacizumab	52 (64.2)
Chemotherapy-based fluoropyrimidine	1 (1.2)
Chemotherapy-based oxaliplatin	25 (30.9)
Chemotherapy-based irinotecan	8 (9.9)
Chemotherapy-based irinotecan and oxaliplatin	18 (22.2)
Aflibercept	1 (1.2)
Chemotherapy-based irinotecan	1 (1.2)
Other^d^	1 (1.2)
**Locoregional treatment,**^e^***n* (%)**	7 (8.6)
Radiotherapy	6 (85.7)
SBRT/IMRT	2 (28.6)
Chemoembolization	1 (14.3)

ECOG, Eastern Cooperative Oncology Group; MSI-H, microsatellite instability-high; IHC, immunohistochemistry; IMRT, intensity-modulated radiotherapy; IQR, interquartile range; SBRT, stereotactic body radiation therapy; *HER2*, human epidermal growth factor receptor-2.

a The tumor stage at diagnosis was not classifiable for one patient from the CONFIDENCE cohort.

b The ECOG performance data at study entry (before first EC cycle) were unavailable for five patients (*n* = 76)

c Molecular characterization was not available for the complete sample of patients: *KRAS* (*n* = 71), *NRAS* (*n* = 70), MSI-H (*n* = 66), *HER2* (*n* = 7).

d Clinical trial: ipilimumab plus nivolumab.

e Multiple response.

### Prior therapy for CRC

Fifty-five (67.9%) patients underwent primary tumor surgical resection [mainly hemicolectomy (40.7%)], and 14 (17.3%) patients underwent metastases resection [mainly peritonectomy (6.2%)]. CRC liver metastases surgical resection was carried out in 3.7% of patients. Twenty (24.7%) patients received adjuvant oncologic treatment and 3 (3.7%) neoadjuvant to surgical procedures in the setting of localized disease.

Locoregional and first-line systemic oncologic treatment received before EC treatment is summarized in Table 1.

### EC treatment

The time since CRC initial diagnosis to EC treatment initiation was 16.2 (9.0-23.5) months, and it was shorter than 18 months for 79.0% of patients. The time since mCRC diagnosis to EC treatment initiation was 9.0 (6.1-16.0) months. Sixteen (19.8%) and 15 (18.5%) patients interrupted encorafenib and cetuximab, respectively, mainly due to toxicity for both of them. At data cut-off, 75 (92.6%) patients had discontinued EC treatment, predominantly because of disease progression (86.7%). Further information is provided in Supplementary Table S1, and a comparative analysis of exposure with another RWD study is provided in Supplementary Table S2, available at https://doi.org/10.1016/j.esmorw.2024.100055.

Two (2.5%) patients underwent surgical intervention for metastatic disease while on EC treatment, achieving R0 resection after one and two interventions, respectively. Locoregional treatment with palliative intention was administered to 4 (4.9%) patients (*n* = 3 stereotactic body radiation therapy/intensity-modulated radiotherapy and *n* = 1 radiotherapy).

### Effectiveness

**Figure 1:**
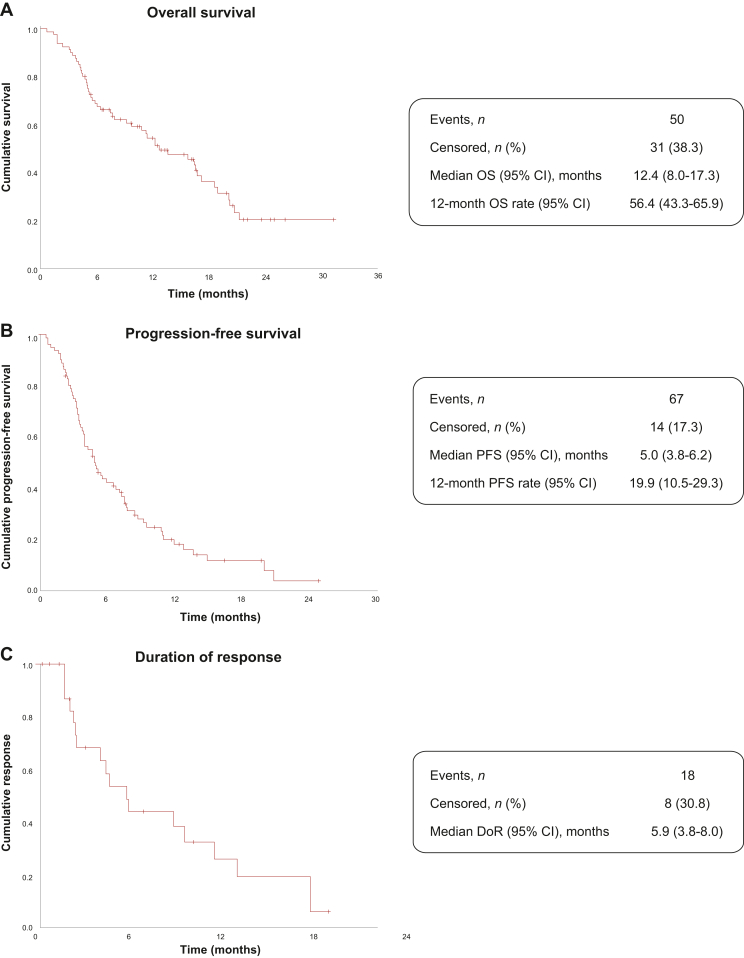
**Survival outcomes.** (A) Overall survival: Kaplan–Meier estimates of cumulative survival. (B) Progression-free survival: cumulative progression-free survival. (C) Duration of response: cumulative response. CI, confidence interval; DoR, duration of response; OS, overall survival; PFS, progression-free survival; TTP, time to progression, which was calculated as events of progression or death due to progression. Since all the death events occurred in the follow-up time were progression events, the TTP calculated is equal to PFS.

The Kaplan–Meier curves for OS and PFS are shown in Figure 1A and B, respectively. With 9.7 months of median follow-up, the median OS was 12.6 (95% CI 8.0-17.3) months, and the 12-month OS rate was 54.6%. The median PFS was 5.0 (95% CI 3.8-6.2) months and PFS rate at 12 months was 19.9%. The median TTP was 5.0 (95% CI 3.8-6.2) months.

The median time to response was 2.2 (2.0-2.5) months. Tumor response with EC treatment was available in 77/81 patients. The ORR was 32.1% (95% CI 22.2% to 43.4%); 25 patients achieved a PR, and 1 patient reached a CR (Table 2). The median DoR was 5.9 (95% CI 3.8-8.0) months (Figure 1C).

**Table 2:** Tumor response and survival outcomes with EC treatment

Tumor response and survival outcomes	*N* = 81
Best response to treatment, *n* (%)	
CR	1 (1.2)
PR	25 (30.9)
SD	27 (33.3)
PD	24 (29.6)
Not evaluable	4 (4.9)
ORR, % (95% CI)	32.1 (22.2-43.4)
DCR, % (95% CI)	65.4 (54.0-75.7)
OS, median (95% CI), months	12.6 (8.0-17.3)
PFS, median (95% CI), months	5.0 (3.8-6.2)

CR, complete response; DCR, disease control rate; ORR, overall response rate; OS, overall survival; PFS, progression-free survival; PR, partial response; SD, stable disease.

### Prognostic factors for PFS and OS

The presence of three or more metastatic lesions, higher values of alkaline phosphatase (AP) and higher neutrophil/lymphocyte ratio (NLR) were found as factors independently associated with shorter OS. Similarly, the presence of three or more metastatic lesions, the presence of liver metastases and higher values of AP and NLR were identified as factors independently associated with shorter PFS (Table 3).

**Table 3:** Univariate and multivariate analysis to evaluate prognostic factors associated to OS and PFS

Characteristics	Overall survival				Progression-free survival			
	Univariate analysis		Multivariate analysis^a^		Univariate analysis		Multivariate analysis^a^	
	HR (95% CI)	*P* value	HR (95% CI)	*P* value	HR (95% CI)	*P* value	HR (95% CI)	*P* value
Age^b^	1.15 (0.65-2.01)	0.636	—	—	0.93 (0.57-1.51)	0.759	—	—
Sex^c^	1.09 (0.62-1.90)	0.769	—	—	1.15 (0.71-1.87)	0.564	—	—
ECOG^d^	6.13 (2.53-14.90)	<0.001	—	—	5.23 (2.15-12.74)	<0.001	—	—
Time from metastatic diagnosis^e^	1.63 (0.81-3.27)	0.170	—	—	1.48 (0.80-2.71)	0.209	—	—
Number of metastatic lesions^f^	3.33 (1.78-6.21)	<0.001	3.69 (1.87-7.27)	<0.001	2.85 (1.58-5.12)	<0.001	1.85 (0.91-3.73)	0.088
Liver metastatic lesion^g^	2.00 (1.12-3.57)	0.019	—	—	2.02 (1.22-3.35)	0.006	2.037 (1.06-3.91)	0.032
Initial metastatic diagnosis^g^	1.66 (0.90-3.03)	0.102	—	—	2.03 (1.19-3.44)	0.009	—	—
Location of primary tumor^h^	1.31 (0.72-2.36)	0.373	—	—	1.01 (0.61-1.66)	0.976	—	—
Surgery of primary tumor^g^	0.51 (0.28-0.90)	0.021	—	—	0.52 (0.31-0.88)	0.015	—	—
*KRAS*^i^	17.05 (1.91-152.52)	0.011	—	—	34.50 (3.13-380.45)	0.004	—	—
*PI3K*^i^	1.47 (0.24-8.88)	0.678	—	—	0.82 (0.15-4.33)	0.811	—	—
*RAS*^i^	22.0 (2.00-242.60)	0.012	—	—	22.0 (1.99-242.60)	0.012	—	—
*HER2*^j^			—	—		0.348	—	—
Positive	0.06 (0.00-3.3 × 10^6^)	0.760	—	—	4.23 (0.26-67.87)	0.308	—	—
Other technique	6380.2 (0.0-1.1 × 10^34^)	0.805	—	—	8.72 (0.40-190.51)	0.169	—	—
MSI^g^	0.43 (0.13-1.41)	0.163	—	—	0.57 (0.22-1.44)	0.232	—	—
CEA level^k^	1.00 (1.00-1.00)	0.001	—	—	1.00 (1.00-1.00)	0.001	—	—
NLR continuous^k^	1.318 (1.16-1.47)	<0.001	1.31 (1.11-1.54)	0.002	1.22 (1.11-1.34)	<0.001	1.29 (1.14-1.46)	<0.001
NLR dichotomic^l^	1.80 (1.00-3.23)	0.049	—	—	2.27 (1.36-3.78)	0.002	—	—
LDH^k^	1.00 (1.00-1.00)	0.459	—	—	1.00 (1.00-1.00)	0.084	—	—
CRP^l^	1.01 (1.00-1.02)	0.018	—	—	1.00 (1.00-1.01)	0.158	—	—
Alkaline phosphatase^l^	1.00 (1.00-1.00)	<0.001	1.00 (1.00-1.00)	<0.001	1.00 (1.00-1.00)	<0.001	1.00 (1.00-1.00)	0.006

CEA, carcinoembryonic antigen; CRP, C-reactive protein; ECOG, Eastern Cooperative Oncology Group; LDH, lactate dehydrogenase; MSI, microsatellite instability; NLR, neutrophil/lymphocyte ratio; *HER2*, human epidermal growth factor receptor-2; *PI3K*, phosphoinositide 3-kinases.

a Only significant variables are shown in the multivariate analysis.

b ≥65 years versus <65 years: reference category: <65 years.

c Male versus female; reference category: male.

d 0-1 versus ≥2; reference category: 0-1.

e <18 months versus ≥18 months; reference category: ≥18 months.

f ≤2 lesions versus ≥3 lesions; reference category: ≤2 lesions.

g Yes versus no; reference category: no.

h Left colon (left or rectum) versus right colon (right or transverse colon); reference category: left.

i Mutated versus wild type (WT); reference category: WT.

j Positive versus negative versus other technique; reference category: negative.

l NLR <3 versus NLR ≥3; reference category: NLR <3.

k Continuous covariates.

Table 4 summarizes the variables of interest in our study along with the two similar studies published to date.

**Table 4:** Baseline characteristics and treatment effectiveness: comparison among studies

	CONFIDENCE cohort *N* = 81	BEACON cohort *N* = 220	Boccaccino et al. cohort *N* = 97
**Patient and disease characteristics**			
Female sex, *n* (%)	41 (50.6)	106 (48.0)	59 (61.0)
Age, median (IQR), years	66.1 (58.9-71.2)	61 (30-91)	68.0 (26.0-85.0)
Location of primary tumor, *n* (%)			
Left sided	31 (38.3)	83 (38.0)	20 (21.0)
Right sided or both sides	50 (61.7)	110 (50.0)	68 (70.0)
ECOG performance status, *n* (%)			
0	28 (36.8)	112 (51.0)	36 (37.0)
1	42 (55.3)	104 (47.0)	48 (50.0)
2	6 (7.9)	4 (2.0)	13 (13.0)
Liver metastases, *n* (%)	44 (54.3)	134 (61.0)	51 (53.0)
≥3 metastases sites, *n* (%)	18 (22.2)	103 (47.0)	41 (42.0)
Treatment exposure, median (IQR), months	4.4 (2.6-7.8)	4.4^a^^,^^b^	4.4^a^^,^^b^
Treatment discontinuation	75 (92.6)	186 (84.5)	106 (80.0)
Reasons for treatment discontinuation			
Progressive disease	65 (86.7)	145 (65.9)	78 (74.0)
Changes in patient condition	1 (1.3)	11 (5.0)	0 (0.0)
Death	2 (2.7)	6 (2.7)	0 (0.0)
Adverse event or failure to tolerate study drug	3 (4.0)	11 (5.0)	4 (4.0)
Physician decision	1 (1.3)	4 (1.8)	0 (0.0)
Patient decision	0 (0.0)	3 (1.4)	0 (0.0)
Dose interruption	NA^c^	2 (0.9)^d^	0 (0.0)
Other^e^	3 (3.7)	4 (1.8)	2 (2.0)
**Effectiveness**			
Best response to treatment, *n* (%)			
CR	1 (1.2)	7 (3.0)	NA
PR	25 (30.9)	36 (16.0)	NA
SD	27 (33.3)	124 (56.0)	61 (46.0)
PD	24 (29.6)	21 (10.0)	NA
Not evaluable	4 (4.9)	32 (15.0)	NA
ORR, % (95% CI)	32.1 (22.2-43.4)	19.5 (14.5-25.4)	17.0^b^
DCR, % (95% CI)	65.4 (54.0-75.7)	NA	65.0^b^
Median OS, (95% CI), months	12.6 (8.0-17.3)	9.3 (8.0-11.3)	7.2 (0.73-1.86)
Median PFS, (95% CI), months	5.0 (3.8-6.2)	4.5 (4.2-5.4)	4.6 (0.74-1.69)

Table 4 has been designed to show an overview of the current context between the three available studies treating advanced mCRC patients with the double combination encorafenib–cetuximab. We have taken into account only the doublet groups from the BEACON clinical trial^19^ and the RWE study conducted by Boccaccino et al.^23^

CI, confidence interval; COVID, coronavirus disease; CR, complete response; DCR, disease control rate; ECOG, Eastern Cooperative Oncology Group; IQR, interquartile range; NA, not available; ORR, overall response rate; OS, overall survival; PD, progressive disease; PFS, progression-free survival; PR, partial response; SD, stable disease.

a The original publication provides the median exposure in weeks (19 weeks).

b 95% CI was not provided.

c Dose interruptions were recorded separately: 16 (19.8%) patients interrupted encorafenib doses and 15 (18.5%) patients interrupted cetuximab doses.

d Other reasons for discontinuation; CONFIDENCE: COVID pneumonia (1.3%), physician decision to primary tumor resection (1.3%) and other non-specified reason (1.3%). BEACON: withdrawal of consent (1.4%), other non-specified reason (0.5%). Boccaccino et al.: treatment-unrelated reasons (2.0%).

### Safety

The incidence of adverse events (AEs) occurring in >5% of patients is shown in Table 5. A total of 216 AEs were reported in 67 (82.7%) patients, and 55 (67.9%) of them reported at least one EC-related AE. Grade 3/4 AEs were present in 22.2% of patients, with acne (3.7%) being the most frequent. EC-related grade 3/4 AEs were present in 11 (13.5%) patients and no grade 5 AEs were registered. Skin-related toxicities were reported in 31 patients (38.3%); of which, 26 (32.1%) were associated to EC.

**Table 5:** Adverse events occurring in >5% of patients

Adverse event^a^	Patients with adverse events, *n* (%)	
	All grades	Grades 3-4^b^
Any	67 (82.7)	18 (22.2)
Asthenia	21 (25.9)	1 (1.2)
Rash/acneiform dermatitis	16 (19.8)	1 (1.2)
Arthralgia	11 (13.6)	1 (1.2)
Acne	9 (11.1)	3 (3.7)
Nausea	8 (9.8)	0 (0.0)
Skin toxicity	6 (7.4)	0 (0.0)
Pyrexia	5 (6.2)	0 (0.0)
Pneumonia	2 (2.5)	2 (2.5)

AEs, adverse events; MedDRA, Medical Dictionary for Regulatory Activities.

a Reported using standard MedDRA dictionary coding.

b No grade 5 AEs were reported.

### Post-EC treatment for mCRC

Approximately half of the patients (50.6%) received a third-line treatment after EC discontinuation. Fourth-line treatment was administered to 12 (14.8%) patients, and 3 (3.7%) patients required five or more lines of treatment. All administered treatments in third line or beyond are presented in Supplementary Table S3, available at https://doi.org/10.1016/j.esmorw.2024.100055.

## Discussion

The CONFIDENCE study provides insight about RW safety and effectiveness of the EC combination for *BRAF*^*V600E*^*-*mutated mCRC patients in Spain. The main clinical outcomes included a PFS of 5.0 months, an OS of 12.6 months, an ORR of 33.8% and a DCR of 68.8%. The most frequent AEs were skin-related toxicities, and the occurrence of grade 3 or worse AEs was 13.5%. In addition, this analysis identified NLR, AP and the presence of three or more metastases sites as potential poor prognostic factors for OS and PFS with the EC combination.

The patient profile was consistent with previous observational and clinical research: female, older than 60 years, with right-sided primary tumor and liver- and peritoneum-dominant disease.^17^, ^18^, ^19^^,^^21^^,^^22^

Table 4 presents a comparison of all variables of interest with the data obtained from similar studies, providing an overview of the baseline characteristics and effectiveness outcomes in patients with *BRAF*^*V600E*^-mutated mCRC receiving EC combination.

The BEACON cohort included patients receiving the EC combinations after progression of one or two lines of treatment. The percentage of patients exposed to one prior line of therapy was 66.0%. Our data reflect that RW patients obtained higher ORR (32.1% versus 19.5%) and longer survival (OS 12.6 versus 9.3 months; PFS 5.0 versus 4.5 months), probably derived from earlier administration of EC.

In a similar retrospective cohort, 97 *BRAF*^*V600E*^-mutated mCRC patients who progressed to one or more lines of treatment received EC, and 63 (65.0%) of them were exposed to one prior line. PFS and OS were 4.6 and 7.3 months, respectively.^23^ Similarly, the survival outcomes seem to be poorer when the cohort of patients has been exposed to more than two lines of treatment, reinforcing the idea that the EC treatment should be offered as soon as possible.

Interestingly, the CONFIDENCE cohort presents a notably lower percentage of patients with more than three metastases’ sites, in comparison with the other cohorts (CONFIDENCE 22.2% versus BEACON 47.0% and Boccaccino et al. 42.0%). This characteristic may also explain the better results obtained in our study.

Our safety outcomes align with those described in literature.^19^^,^^24^ Skin-related AEs (43.2%) were the most frequent, coinciding with the BEACON CRC study population, which reported a majority of dermatological toxicities (75.5%).^25^ In summary, our RW cohort reported less AEs and less grade ≥3 AEs, in contrast to the BEACON trial cohort. This deviation may be derived from differences in the recording and reporting of AEs between the clinical practice and the clinical trial. Furthermore, this suggests that an early administration of the EC improves its safety and tolerability, leading to a better disease-related quality of life and higher possibilities to receive further lines of treatment.

NLR has been proved to be a valuable prognostic factor associated to tumor progression in CRC.^26^, ^27^, ^28^ In line with these results, our data suggest that higher values of NLR and AP are related to shorter OS and PFS for *BRAF*^*V600E*^-mutated mCRC patients. Additionally, we found that the presence of three or more metastatic lesions and metastasis in the liver were associated with shorter PFS. Nevertheless, our study did not identify other variables that have been reported as predictors of OS and PFS in mCRC, for instance, Eastern Cooperative Oncology Group performance status, the presence of peritoneal metastasis, a primary tumor surgery, etc.^23^^,^^29^ Even though the association of gene mutations with poorer OS or PFS in comparison to WT genotypes has been previously reported,^30^^,^^31^ we did not find any significant association between *HER2* (human epidermal growth factor receptor-2), *RAS*, *KRAS* or *PI3K* (phosphoinositide 3-kinases) mutations and poorer OS or PFS in this study. In summary, there is still a lack of consensus in determining clear and valuable prognostic factors and further research is necessary to clarify this aspect.

This study presents the limitations related to an observational retrospective investigation, especially in the detection and reporting of AEs and serious AEs (SAEs). Even though safety monitoring is not mandatory for retrospective studies, we focused our efforts in collecting all possible safety information to elaborate a complete safety analysis of EC administration to a RW cohort of patients. Although we provide extensive information, the safety analysis must be interpreted cautiously because potentially occurring AEs and SAEs may not be included. The selection of patients followed up for at least 5 months may imply a selection bias and impact the efficacy data, since patients with shorter follow-up could not be included in the analysis. Furthermore, other design-related limitations such as the heterogeneity in the time to imaging test in each hospital may affect the results. Given the recent approval of the EC combination and the lack of data published since approval, we should consider these results with caution because of the sizable differences in design, treatments, intention of the analysis, location of the primary tumor, etc., among the studies discussed in this work. Despite these limitations, our research provides novel and valuable data from a RW setting. To our knowledge, this is one of the first studies involving *BRAF*^*V600E*^-mutated mCRC patients treated with EC combination in routine practice in the world, and the first conducted in Spain. Our results provide essential information about the reproducibility of the results obtained from the introduction of second-line EC in the current clinical practice, and suggest even better results in survival, response and safety when it is administered early.

## Conclusion

The efficacy and safety outcomes proved during the BEACON trial have been confirmed with this analysis, with even better outcomes observed if the treatment is administered before the third line. This analysis contributes novel, retrospective RWD which is of the utmost importance to understand the actual impact of the treatment in patients treated under clinical practice conditions and out of the clinical trial-controlled conditions.
