# Supplementary material for: Safety and efficacy of encorafenib–cetuximab combination in BRAFV600E-mutated metastatic colorectal cancer: real-world evidence from the CONFIDENCE Spanish multicenter study
Source: ESMO Real World Data Digit Oncol. 2024 Jul 16;5:100055. doi: 10.1016/j.esmorw.2024.100055 (PMC12836585; doi:10.1016/j.esmorw.2024.100055)
Supplement: Supplemental Material [file mmc1.docx]

# **Supplementary material**

**Table S1. EC treatment exposure and modification**

| **Treatment exposure and modifications** | **N=81** | |
| --- | --- | --- |
|  | **Encorafenib** | **Cetuximab** |
| **Number of cycles,** median (IQR) | 6.0 (4.0-13.0) | 6.0 (4.0-14.5) |
| **Treatment duration (months),** median (IQR) | 4.4 (2.6-7.8) | 4.4 (2.6-7.6) |
| **Dose reduction,** n (%) | 20 (24.7) | 7 (8.6) |
| Toxicity, n (%) | 17 (85.0) | 6 (85.7) |
| Skin-related, n (%) | 4 (23.5) | 5 (83.3) |
| Worsening functional status, n (%) | 1 (5.0) | 0 (0.0) |
| Adverse events, n (%) | 0 (0.0) | 1 (14.3) |
| Other reasons, n (%) | 2 (10.0) | 0 (0.0) |
| **Return to starting dose,** n (%) | 1 (5.0) | 0 (0.0) |
| **Treatment interruptions,** n (%) | 16 (19.8) | 15 (18.5) |
| Toxicity, n (%) | 9 (56.3) | 8 (53.3) |
| Skin-related, n (%) | 4 (44.4) | 6 (75.0) |
| Worsening functional status, n (%) | 1 (6.3) | 1 (6.7) |
| Adverse events, n (%) | 1 (6.3) | 1 (6.7) |
| Due to other reasons, n (%) | 3 (18.9) | 4 (26.8) |
| **Overall treatment discontinuation** | 75 (92.6) | |
| Progressive disease | 65 (86.7) | |
| Changes in patient condition | 1 (1.3) | |
| Adverse event or failure to tolerate study drug | 3 (4.0) | |
| Death | 2 (2.7) | |
| Physician decision | 1 (1.3) | |
| COVID-pneumonia | 1 (1.3) | |
| Other ^a^ | 2 (2.6)^ǂ^ | |

IQR, Interquartile range; n, number of patients

Other reasons for discontinuation: physician decision to primary tumor resection (1.3%), and other non-specified reason (1.3)

**Table S2. Comparative of treatment exposure between the CONFIDENCE cohort and the RWE cohort studied by Boccaccino and cols.**

|  | **CONFIDENCE cohort**  **N=81** | | ***Boccaccino and cols.***  **N=133^a^** | |
| --- | --- | --- | --- | --- |
|  | **Encorafenib** | **Cetuximab** | ***Encorafenib*** | ***Cetuximab*** |
| **Number of cycles,** median (IQR) | 6.0 (4.0-13.0) | 6.0 (4.0-14.5) | 4.0 (1-19) ^b^ | |
| **Treatment duration (months),** median (IQR) | 4.4 (2.6-7.8) | 4.4 (2.6-7.6) | 4.4 ^b,c^ | |
| **Dose reduction,** n (%) | 20 (24.7) | 7 (8.6) | 18 (14.0) | 5 (4.0) |
| Toxicity, n (%) | 17 (85.0) | 6 (85.7) | 0 (0.0) | 0 (0.0) |
| Skin-related, n (%) | 4 (23.5) | 5 (83.3) | 0 (0.0) | 0 (0.0) |
| Worsening functional status, n (%) | 1 (5.0) | 0 (0.0) | 0 (0.0) | 0 (0.0) |
| Adverse events, n (%) | 0 (0.0) | 1 (14.3) | 18 (100.0) | 0 (0.0) |
| Other reasons, n (%) | 2 (10.0) | 0 (0.0) | 0 (0.0) | 0 (0.0) |
| **Return to starting dose,** n (%) | 1 (5.0) | 0 (0.0) | NA | NA |
| **Treatment interruptions,** n (%) | 16 (19.8) | 15 (18.5) | 47 (35.0) | 34 (26.0) |
| Toxicity, n (%) | 9 (56.3) | 8 (53.3) | 0 (0.0) | 0 (0.0) |
| Skin-related, n (%) | 4 (44.4) | 6 (75.0) | 0 (0.0) | 0 (0.0) |
| Worsening functional status, n (%) | 1 (6.3) | 1 (6.7) | 0 (0.0) | 0 (0.0) |
| Adverse events, n (%) | 1 (6.3) | 1 (6.7) | 28 (59.6) | 17 (50.0) |
| Other reasons, n (%) | 3 (18.9) | 4 (26.8) | 19 (40.4) ^d^ | 17 (50.0) ^e^ |

1. Data presented in this table is calculated from the total population of patients (N=133), including doublet and triplet groups.
2. Data available for the EC combination. No individual data was published.
3. The original publication provides the median exposure in weeks (19 weeks). 95% CI was not available.
4. Non-specified reasons
5. Treatment-unrelated reasons.

**Table. S3 Therapies received after EC discontinuation for mCRC**

| **Therapies received after EC discontinuation** | **Third line**  **(N =41)** | **Fourth line and beyond**  **(N=12) ^d^** |
| --- | --- | --- |
| **Chemotherapy based (only)** |  |  |
| Chemotherapy based irinotecan | 8 (19.5) | 1 (8.3) |
| Chemotherapy based oxaliplatin | 2 (4.9) | 3 (25.0) |
| Chemotherapy based Trifluridine/Tipiracil | 3 (7.3) | 2 (16.7) |
| **Anti-angiogenic based** |  |  |
| Chemotherapy based aflibercept | 4 (9.8) | - |
| Chemotherapy based bevacizumab | 18 (43.9) | 3 (25.0) |
| **Regorafenib** | 2 (4.9) | 1 (8.3) |
| **Encorafenib + Cetuximab ^a^** | 1 (2.4) | - |
| **Other ^b,c^** | 3 (7.3) | 2 (16.7) |

1. Administered as 3^rd^ line after progression.
2. Other third line treatments: pembrolizumab (1 patient), clinical trials (2 patients)
3. Other fourth line treatments: clinical trials (2 patients)

Two patients received 5^th^ lines of treatment and one patient received 6^th^ lines of treatment.
